# Cross-species transmission, evolution and zoonotic potential of coronaviruses

**DOI:** 10.3389/fcimb.2022.1081370

**Published:** 2023-01-06

**Authors:** Qian Li, Taif Shah, Binghui Wang, Linyu Qu, Rui Wang, Yutong Hou, Zulqarnain Baloch, Xueshan Xia

**Affiliations:** ^1^ Faculty of Life Science and Technology, Kunming University of Science and Technology, Kunming, Yunnan, China; ^2^ Affiliated Anning First People’s Hospital, Kunming University of Science and Technology, Kunming, China; ^3^ The First Affiliated Hospital & Clinical Medical College, Dali University, Dali, Yunnan, China

**Keywords:** coronaviruses, epidemiology, evolution, cross-species transmission, zoonosis

## Abstract

Coronaviruses (CoVs) continuously evolve, crossing species barriers and spreading across host ranges. Over the last two decades, several CoVs (HCoV-229E, HCoV-NL63, HCoV-HKU1, HCoV-OC43, SARS-CoV, MERS-CoV, and SARS-CoV-2) have emerged in animals and mammals, causing significant economic and human life losses. Due to CoV cross-species transmission and the evolution of novel viruses, it is critical to identify their natural reservoiurs and the circumstances under which their transmission occurs. In this review, we use genetic and ecological data to disentangle the evolution of various CoVs in wildlife, humans, and domestic mammals. We thoroughly investigate several host species and outline the epidemiology of CoVs toward specific hosts. We also discuss the cross-species transmission of CoVs at the interface of wildlife, animals, and humans. Clarifying the epidemiology and diversity of species reservoirs will significantly impact our ability to respond to the future emergence of CoVs in humans and domestic animals.

## Brief introduction

Coronaviruses (CoVs) are positive-sense, linear, non-segmented, single-stranded RNA viruses (Shah et al., 2022). These viruses are grouped into the order *Nidovirales*, family *Coronaviridae*, and subfamily *Coronavirinae*, with a genome size of about 27~32 kb. The subfamily has been further classified into genera: alpha Human CoVs (HCoV-229E and HCoV-NL63); beta HCoVs (severe acute respiratory syndrome (SARS)-CoV, Middle East respiratory syndrome (MERS)-CoV, HCoV-HKU1, and HCoV-OC43); gamma-CoVs and delta-CoVs (Zhang et al., 2018). These viruses cause respiratory, digestive, and even nervous system disorders in humans, animals (rats, pigs, turtles, whales, etc.), and poultry (Dijkman et al., 2013; Sipulwa et al., 2016; Mulabbi et al., 2021). The average incubation period of this disease is about 5.2 days (Li et al., 2020a), exhibiting manifestations in humans, including respiratory tract disorders, sneezing, dry cough, sore throat, etc. In addition, COVID-19 patients experienced diarrhea, while SARS or MERS cases have a low probability of developing such disease symptoms (Lee et al., 2003; Assiri et al., 2013).

SARS-CoV-2 has given a 5% mortality rate to the current global pneumonia compared to SARS-CoV (10% mortality) and MERS-CoV (34% mortality) (Gabutti et al., 2020; Wang et al., 2020b). Until the SARS emergence in China in 2002–2003, CoVs had been of greater concern to agriculture than public health. During the SARS-CoV outbreak, numerous scientific efforts have focused on identifying and characterizing additional CoVs in wildlife and mammals. Consequently, several novel CoVs have been reported in bats, and poultry over the past two decades (Walsh et al., 2013). The current SARS-CoV-2 outbreak has again put CoVs in the global spotlight and is marked as the most highly pathogenic human virus after SARS-CoV and MERS-CoV. Indeed, many unidentified CoVs may circulate in bats (natural reservoirs) before transmission to humans, animals, or poultry *via* intermediate hosts (Zhu et al., 2022). Therefore, long-term research efforts will instigate the exploration of pathogenic HCoVs that may provide an essential theoretical basis for preventing new outbreaks in the future.

The current review discusses CoVs’ taxonomic, phylogenetic, and epidemiological characteristics. We also highlighted the evolution of CoVs that co-exist with their hosts for long periods to cause persistent infection. Moreover, we describe CoV’s host cellular receptors, their entry mechanisms into the host cell. Finally, we discuss CoVs-vulnerable hosts and cross-species transmission.

## Taxonomic and diversity of CoVs

The International Committee on Taxonomy of Viruses (ICTV) first proposed the order *Nidovirales* (in Latin, *nido* means nest) in 1996 (Pringle, 1996), which primarily comprises the *Coronaviridae* and *Arteriviridae* viral families (Pringle, 1996). Later, improved detection techniques and metagenomics included more novel viruses in the *Nidovirales* order (Ge et al., 2012; Shi et al., 2018), which profoundly changed the Virus Classification System. Currently, eight suborders (*Arnidovirineae*, *Abnidovirineae*, *Cornidovirineae*, *Nanidovirineae*, *Mesnidovirineae*, *Monidovirineae*, *Ronidovirineae*, and *Tornidovirineae*) are included in the *Nidovirales* order (Walker et al., 2019). These suborders comprise fourteen families, twenty-five subfamilies, thirty-nine genera, sixty-five subgenera, and one hundred and nine species. Based on genotypic and serological characteristics, CoVs were further classified into alpha, beta, gamma, and delta genera. A study reported seven different CoVs, i.e., alpha CoVs (HCoV-229E and HCoV-NL63) and beta CoVs (HCoV-HKU1, HCoV-OC43, SARS-CoV, MERS-CoV, and SARS-CoV-2), infecting humans (Kesheh et al., 2022; Li et al., 2022a). Genera alpha and beta CoVs included subgroups A, B, C, and D that infect humans and animals (cats, cows, dogs, horses, mice, pigs, etc.), whereas gamma and delta infect birds along with other mammals (Kesheh et al., 2022; Li et al., 2022a). Due to their high pathogenicity, alpha- and beta HCoVs have attracted more attention (Schoeman et al., 2021). Gamma and delta avian CoVs infect chicken, duck, goose, pigeon, sparrow, and turkey with a 5% infection rate (Wille and Holmes, 2020). Due to their wide distribution and strong migratory characteristics, bats may increase the risk of disease transmission to animals or humans. Recently, with the occurrence of SARS-CoV2, relevant departments have strengthened safe bat migration and detection mechanisms.

## Epidemiology of CoVs

SARS, the first emerging epidemic of the twenty-first century (lasting approximately six months), first appeared in China and quickly spread to other countries, including Europe and America. All the SARS cases experienced high-grade fever (>100.3°F), headache, aches, diarrhea, feeling uncomfortable, cough, respiratory disorders, and pneumonia (in severe cases). SARS-CoV is most commonly transmitted *via* inhalation of respiratory droplets from an infected person to healthier ones or by touching virus-contaminated surfaces. CDC’s collaboration with WHO and other global partners addressed SARS’s global crisis by providing emergency health services, deploying medical specialists to assist with on-site investigations, and providing extensive clinical testing for SARS patients (CDC, 2022). Apart from the SARS-CoV outbreak, MERS-CoV reportedly infect human with similar clinical symptoms to SARS-CoV (de Wit et al., 2016).

The epidemic of animal and poultry CoVs causes significant economic challenges, and their transmission may threaten the human population due to their close association. In 1984, McNulty and his team detected Bovine CoV (BCoV) in calf lungs suffering from pneumonia, diarrhea, and respiratory disorders in America, Europe, Asia, and Africa (Zhu et al., 2022), revealing CoV transmission among poultry and birds. The transmission of viruses from poultry to birds represents a new paradigm at the livestock-wildlife interface. Researchers discovered three avian CoV species in 297 wild birds in Egypt between early 2014 and late 2015. The genetic characterization revealed a close relationship with the country’s most commonly used live-attenuated vaccines, highlighting extensive vaccine strain spillover in poultry (Rohaim et al., 2017). In 2015, researchers in Italy investigated the genetic diversity of CoVs in quail, pheasant, and partridge. Avian CoV (gamma CoVs) were found in quail that had not been immunized against the Massachusetts serotype of the Infectious Bronchitis Virus (IBV) and in pheasants that had been vaccinated with IBV. Avian CoVs found in quail and pheasants were linked to IBV-793B and IBV types based on gene sequence homologies encoding spike proteins. *RdRp* nucleotide sequence analyses revealed quail susceptibility to Delta CoVs, implying that avian CoVs from quail and pheasants share spike gene homology with chicken IBV (Torres et al., 2017). A study reported a novel avian IBV in commercial chicken in Brazil (Fraga et al., 2013) and domestic peafowl in Guangdong Province, China (Liu et al., 2005). In Poland, the overall CoV prevalence in wild birds was 4.15%, with orders *Anseriformes* (3.51%) and *Charadriiformes* (5.59%) being the primary reservoirs. Gamma CoVs were more frequently detected (3.5%) in six bird orders (*Anseriformes*, *Charadriiformes*, *Columbiformes*, *Galliformes*, *Gruiformes*, and *Passeriformes*) than delta CoVs (0.7%), which requires a taxonomic update. CoV detection in *Anseriformes* (3.51%) and *Charadriiformes* (3.1%) belonged to the subgenera *Igacovirus* and *Brangacovirus*, respectively. Most of these were *igacoviruses* belonging to the duck-CoV-2714 and two avian CoV-9203 phylogenetic groups. In addition, a porcine delta CoV (PDCoV) HKU15, was identified in Hong Kong in 2012 (Woo et al., 2012), infecting pig intestinal epithelia and causing severe diarrhea and vomiting (Ma et al., 2015). Several PDCoV strains were later detected in the United States (Alhamo et al., 2022), Canada (Ajayi et al., 2018), South Korea (Chung et al., 2017), Thailand (Janetanakit et al., 2016), China (Huang et al., 2020a), Japan (Suzuki et al., 2018), Vietnam (Le et al., 2018), etc., posing a severe threat to humans population associated with the swine industry. So far, all members of the delta CoV genus have been found in birds (Saeng-Chuto et al., 2017), implying that birds serve as the natural reservoir for these viruses (Jung et al., 2015; Ma et al., 2015). The close association of the PDCoV genome with the sparrow CoV-HKU17 genome might be attributed to the recombination between Bulbul-CoV HKU11 (Lau et al., 2018). Therefore, identifying novel pathogenic PDCoV may provide opportunities to define the underlying mechanism that allows viruses to cross the host-range barrier. The close interaction of swine and humans endangers public health, as evidenced by the swine-originated influenza virus outbreak (Smith et al., 2009). The prevention and control strategies for PDCoV cross-species transmission among wildlife and livestock demand further investigation to unveil the future dispersal history of CoVs, evolution, and their cross-species transmission.

All highly pathogenic alpha- and beta HCoVs and animal CoVs primarily originate in bats before transmission to humans or animals. For example, SARS-CoV originated in bats (Graham and Baric, 2010), while MERS-CoV originated in camels before being transmitted to humans (Dudas et al., 2018). The current pathogenic novel SARS-CoV-2, first reported in Wuhan, China, is responsible for a significant public health crisis and global economic and social challenges (Gabutti et al., 2020; Zhu et al., 2020). Many of the first COVID-19 patients claimed to have visited an animal market in Wuhan, implying that the virus may have been transmitted to them by animals at the market (Zhou et al., 2020). Although the exact route of SARS-CoV-2 transmission from bat reservoirs to humans is unknown (Lu et al., 2020; Zhou et al., 2020), recent evidence points to raccoons as the intermediate mammalian host between bats and humans (Worobey et al., 2022). Because of its high contagiousness, SARS-CoV-2 spreads rapidly across countries, claiming substantial life losses (WHO, 2020). The emergence of SARS-CoV in 2002, MERS-CoV in 2012, and the current SARS-CoV-2 outbreak in 2019 provide evidence for future CoV outbreaks.

## Evolution of CoVs

Adaptive evolution is the only way for CoVs to co-exist with their hosts over a long period for successful infection. CoVs have a high error rate of replication (much higher than DNA viruses), resulting in a high mutation rate because they are single-stranded RNA compared with double-stranded DNA structures. Almost all CoVs originating from bats cause various diseases in poultry, animals, and humans (Su et al., 2016; Alluwaimi et al., 2020). Before SARS-CoV emergence, the two HCoV prototypes (HCoV-OC43 and HCoV-229E) were primarily associated with the common cold (Weiss and Navas-Martin, 2005). In contrast, the HCoV variants HCoV-NL63 and HCoV-HKU1 (Zhou et al., 2020) were associated with mild respiratory and enteric diseases in humans (Liu et al., 2021). Bats originated SARS-CoV, which developed in civet cats (as an intermediate host) (Banerjee et al., 2020), and MERS-CoV is the most infectious HCoV that causes severe infection (Yin and Wunderink, 2018). Given the prevalence of the SARS-like virus in bats and their vast genetic diversity, coexistence, and frequent CoV recombination, new mutations are expected in the future. CoVs, cross-host species, and barriers spread over a wide area over time. In rare cases, viruses transmit across host species for infection. In recent years, the chance of cross-species transmission of mammalian viruses has increased due to increased human-animal contact. The viruses crossing the animal-human species barrier are the avian influenza virus, Hantavirus, hemorrhagic fever virus, insect-borne virus, monkeypox virus, Nipa and Hendra viruses, and SARS-CoVs (Louz et al., 2005; Olival et al., 2017).

CoVs have low mutation rates and high degrees of genetic diversity (Liu et al., 2017) compared to other single-stranded RNA viruses (Dudas and Rambaut, 2016; Su et al., 2016). The first reports of CoV recombination in the 1980s focused on infections with different murine mouse hepatitis viruses (Keck et al., 1988). CoV genome analyses from natural infections have yielded evidence that recombination, particularly between divergent CoVs within individual subgenera, significantly contributes to CoV evolution (Lee and Jackwood, 2000). For example, a complex recombinant has been observed between the Alpha CoV-1 (canine CoV), which primarily infects dogs (Lednicky et al., 2022), the feline CoV, which infects cats (Herrewegh et al., 1998), and the transmissible gastroenteritis virus from swine, which infects pigs (Decaro et al., 2009). Copy-choice is the most common CoVs recombination mechanism in which a viral RNA-dependent RNA polymerase (RdRp) is interrupted during replication, drops off the copying RNA template, and re-engages with a different RNA template at a homologous position before replication (Cheng and Nagy, 2003). During replication, such template switches (recombination breakpoints) transform recombinant daughter genomes with different sequences derived from parental genomes, most likely giving viruses more evolutionary options than mutation alone (Simon-Loriere et al., 2009). Many newly arising mutations within the genomes of genetically compact CoV are expected to have negative fitness consequences, as are mutations that occur between genetically divergent viruses (Drummond et al., 2005). Recombination frequently disrupts favorable co-evolved interactions (referred to as epistatic interactions) within the genome by transferring nucleotide sequences into genomic backgrounds with which they did not co-evolve. For example, recombination (pairing nucleotides to form biologically functional genomic secondary structures) could disrupt epistatic interactions (Martin et al., 2005; de Klerk et al., 2022). However, because recombination generally occurs between fully functional genomes, the range of potential negative fitness consequences should be less extreme than that of newly arising mutations (Drummond et al., 2005). Recombination frequency among CoVs is so high that it occurs without external stress and becomes the dominant virus population (Lai, 1992; Simon-Loriere and Holmes, 2011).

**Figure 1:**
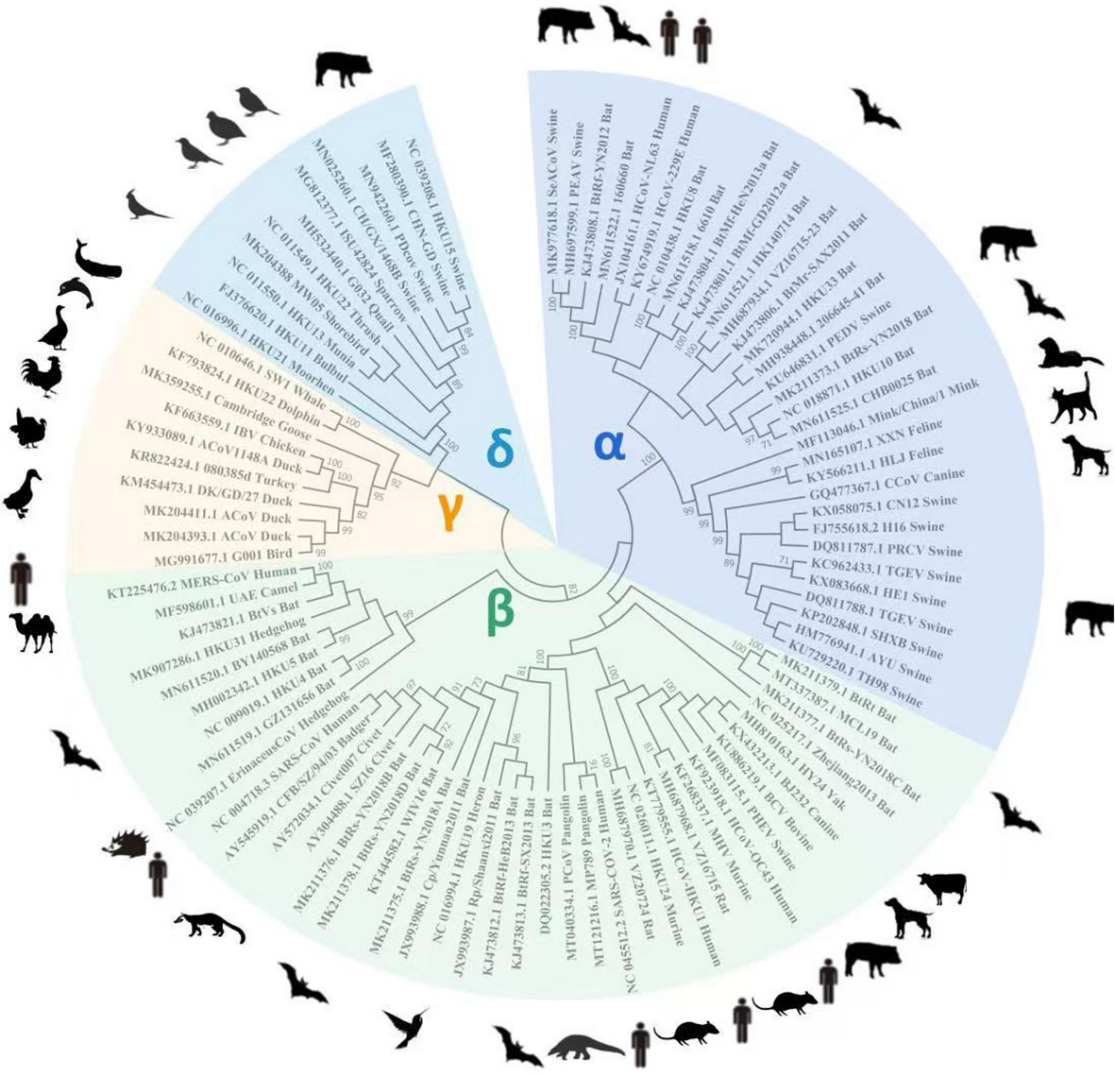
For *RdRp* gene homology analysis, 93 known *RdRp* gene amino acid sequences were retrieved from the NCBI database to construct a phylogenetic tree using Mega X software. The tree shows alpha and beta CoVs genera had the highest RdRp amino acid sequence homology, followed by gamma and delta viruses.

A total of 93 different CoV RdRp protein sequences (that exhibited ~90% similarity) were retrieved from the NCBI database to construct a phylogenetic tree. All the RdRp protein sequences were compared for similarities based on their amino acid residues. CLUSTALW-aligned RdRp sequences were used to construct a tree for understanding RdRp homology from different CoVs. Bats and pigs are the most important natural reservoirs for alpha, beta, gamma, and delta CoVs. The phylogenetic tree shows that alpha and beta CoV genera had high sequence similarity, followed by gamma and delta (**Figure 1**).

CoVs are positive-sense single-stranded RNA viruses with giant RNA genomes (ranging from 27 to 32 kb in length) capable of trans-species movement, as evidenced by the SARS-CoV outbreak (Drosten et al., 2003; Ksiazek et al., 2003). In addition, the emergence of MERS-CoV in 2012 shows that CoVs continue to cause severe human disease (Zaki et al., 2012). The discovery of 3′–5′ exoribonuclease activity within the non-structural nsp14 exoribonuclease, critical for CoV high-fidelity replication, calls into question the long-held belief that RNA viruses cannot proofread and raises the possibility of an entirely new model for how these viruses regulate replication fidelity. RNA viruses rely primarily on low *RdRp* gene fidelity to facilitate their adaptation to host environments (Domingo et al., 2012). The average mutation rate of RNA viruses is estimated to be around one mutation per genome per round of replication (Drake and Holland, 1999; Malpica et al., 2002). Thus, allowing for enormous population diversity, the low fidelity of RdRp-mediated replication imposes constraints on the maintenance of genomic integrity, theoretically limiting the size of RNA virus genomes to around 15 kb in length (Domingo et al., 2012). Large population sizes, rapid replication cycles, resistance to mutations, recombination, and compact genomes are some of the mechanisms that RNA viruses have evolved to partially circumvent these constraints (Graci et al., 2012; Lauring et al., 2013). These re-assorting mutations generate novel CoV phenotypes with expanded characteristics (Forni et al., 2017). For example, CoV genomic RNA plasticity provides numerous benefits, including the virus’s rapid adaptability to unusual host species with increased virulence. According to scientific evidence, almost all CoVs that infect humans are primarily derived from bats; thus, bats play an essential role in their evolution toward new host species. The jumping of alpha- and beta CoVs from bats to other mammals resulted in introducing these viruses into new hosts (Woo et al., 2012). This “jumping” phenomenon is common among CoVs (Tong et al., 2009; Anthony et al., 2017; Mulabbi et al., 2021), but the underlying molecular mechanism for novel phenotypes transferring to new hosts requires further investigation.

To track the outbreak history and evolution, the CoV infection rate among 1016 diarrheal cattle in Heilongjiang Province, China, was 15.45% in 2020, significantly correlated with age, breeding type, etc. (Lv et al., 2021). A new IBV isolate was isolated from vaccinated chicken in Guangxi, China, in 2016, indicating constant virus evolution and emphasizing the importance of monitoring new IBVs (Lv et al., 2021). In 2019, 3160 poultry samples from 14 Chinese provinces were tested for CoVs. Of the total analyzed samples, 593 were positive for avian CoVs, including 485 avian IBVs, 72 duck CoVs, and 36 pigeon CoVs, demonstrating distinct CoVs in the country. The current study demands more research into these viruses to better understand their cross-species transmission and clinical significance. (Li et al., 2021). According to a study, the avian IBV outbreak in southern China from 1985 to 2017 experienced significant changes in genetic diversity and genotypes, indicating the importance of a long-term virus monitoring system and the urgency of developing new vaccines to combat emerging IBV strains (Fan et al., 2019). More CoV monitoring and research are required to understand virus diversity and evolution better.

## CoVs receptor and host cellular entry mechanism

The spike (S) protein, composed of approximately 1273 amino acids and 150~200 kDa, shows varying degrees of conservation among the CoVs (Yadav et al., 2021). Any conformational changes in S domains may alter viral pathogenesis and host cellular entry mechanisms (Aoe, 2020; Huang et al., 2020b). Following synthesis, the S protein forms a homotrimeric assemblage that contains several domains, including a receptor binding site, etc., that interact with the angiotensin-converting enzyme-2 (ACE2) host receptor (Casalino et al., 2020; Moreira et al., 2020). ACE2 is a type I transmembrane protein found in the kidney, heart, intestines, and lungs. It aids in regulating the renin-angiotensin system by balancing angiotensin and protecting against lung injury and pulmonary hypertension. SARS-CoV, HCoV-NL63, and SARS-CoV-2 use the host cellular ACE2 receptor to enter the host cell cytoplasm (Hofmann et al., 2005; Wan et al., 2020). The virus’s binding to the host surface reduces the ACE2 expression level, resulting in pulmonary injury and even death in severe cases (Ge et al., 2013). In addition, dipeptidyl peptidase-4 (DPP4), a conserved glycoprotein expressed in the kidney, respiratory tract, immune cells, etc. (Danta, 2020), helps in MERS-CoV surface receptor function and mediates viral entry into the host cell cytoplasm (Ghosh et al., 2021).

TMPRSS2 is another host cellular protease generally expressed in the epithelium of the respiratory, digestive, and reproductive tracts (David et al., 2020), priming SARS-CoV-2 S attachment with the host cell. To begin the proteolytically active fusion process, TMPRSS2 cleaves the proximal S2 subdomain, exposing the fusion peptide (Dong et al., 2020). In addition, the virus uses the S1 to bind to the host, triggering the effects of TMPRSS2 on the S cleavage and priming membrane fusion for viral entry (Djomkam et al., 2020; Ju et al., 2020; Walls et al., 2020). In addition to TMPRSS2, members of the C-type lectin mediate viral entry into the host cell to initiate pathogenesis (Amraei et al., 2021). Moreover, the metabotropic glutamate receptor (mGluR2) is a GPCR-class glycoprotein expressed on presynaptic and postsynaptic cells in the CNS (Singh et al., 2021). To mediate SARS-CoV-2 internalization, MGluR2 interacted directly with the virus S protein. In contrast, SARS-CoV-2 penetration was reduced by knocking out mGluR2, and the virus binding capability remains stable (Wang et al., 2021a). Unlike mGluR2, which directly interacts with the SARS-CoV-2 S receptor, AXL specifically interacts with the SARS-CoV-2 N-terminal domain (Wang et al., 2021b) for penetration into the host cell. This virus surface protein also shields the pathogen from antibody neutralization (Gu et al., 2022; Hoffmann and Pöhlmann, 2022). CEACAM1 is another transmembrane protein belonging to the carcinoembryonic antigen family of the immunoglobulins that acts as a receptor for CoVs. The binding of CoV S to the CEACAM1a domain causes a conformational change in the S1 domain, facilitating virus entry into the host cell (Haan et al., 2006). In a study, molecular modeling and docking simulation addressed the interaction between CD13-related domains and the SARS-CoV S receptor. The binding of the SARS-CoV S protein’s D757-R761 motif to the P585-A653 domain of CD13 was simulated, implying that CD13 could be a SARS-CoV S receptor associated with SARS infection (Yu et al., 2003). Some of the CoV species and different host cellular receptors are shown in **Table 1**.

**Table 1:** Characteristics of host cellular receptors for various CoVs.

Host receptors	Expression sites	Normal function	CoV types	Ref
ACE2	The small intestine, duodenum, heart, kidney, gall bladder testis	Regulate vasoconstriction and blood pressure	A receptor for the SARS-CoV/SARS-CoV-2/HCoV- hCoV-NL63	(Wan et al., 2020)
DPP4/CD26	Intestines, kidney, placenta	Glucose and insulin metabolism, as well as immune regulation	A receptor for MERS-CoV/bat-CoV HKU4	(Ng et al., 2022)
APN/ANPEP/CD13)	Intestines	Promote angiogenesis, tumor growth, metastasis	A receptor for the HCoV-229E/PEDV/TGEV/PEAV/PRCV/Canine CoV/FIPV	(Yu et al., 2003)
CEA cell adhesion molecule 1a (CEACAM1a)	Colon, large intestine, small intestine, duodenum	Signaling receptor binding, virion binding, virus receptor activity	A receptor for MHV	(Hemmila et al., 2004)
9-0-Acetylated Sialic Acid	Intestines	Adhesion intercellular, and angiogenic, inhibit the tumor growth	A receptor for the HCoV-OC43/HCoV-HKU1/TPHEV/BCoV/FIPV	(Hulswit et al., 2019)
TMPRSS2	Prostate, stomach, colon, duodenum	Biological processes such as digestion, inflammatory responses, and so on	Interacts with ACE2 and initiates membrane fusion	(Dong et al., 2020)
CD209L	Placenta, lymph node, small intestine, urinary bladder, gall bladder, and duodenum	Encodes a C-type lectin that functions in cell adhesion and pathogen recognition.	Recognize the SARS-CoV	(Amraei et al., 2021)
mGluR2	Brain, testis	Cognitive disorders, drug addiction, psychosis, schizophrenia, anxiety, cerebral ischemia, and epilepsy2	The ectodomain of mGluR2 interacts with ACE2 and is essential for SARS-CoV-2 internalization	(Singh et al., 2021)
AXL	Spleen, endometrium, placenta, lung	Cellular functions, including growth, migration, aggregation, and anti-inflammation	Cooperates with ACE2 to mediate SARS-CoV-2 attachment and entry	(Dagamajalu et al., 2021)
KREMEN1	Skin, esophagus, ovary, heart, testis, colon	A functional receptor for Coxsackievirus A10	Alternative receptor for SARS-CoV-2	(Hoffmann and Pöhlmann, 2022)
ASGR1	Liver	Facilitate multiple viral infections, including hepatitis B	Promote SARS-CoV-2 infection	(Gu et al., 2022)

## CoVs-susceptible hosts

Several CoVs have been confirmed to be primarily hosted by bats, rodents, porcine, mice, birds, etc. (de Wilde et al., 2018). Alpha- and beta CoVs are primarily found in mammals, whereas gamma and delta CoVs are mostly found in birds. Identifying virus-carrying hosts will be critical to disease prevention and control strategies. Humans are vulnerable to at least seven types of human CoVs (HCoV-229E, HCoV-NL63, HCoV-HKU1, HCoV-OC43, SARS, MERS, and SARS-CoV-2), primarily transmitting through respiratory droplets, direct contact with infected people, animals, body fluids, or touching contaminated surfaces (Milewska et al., 2014; Ye et al., 2020). CoV-29E or HCoV-OC43 causes approximately 30% of common colds in humans, particularly children (Dijkman et al., 2008; Walsh et al., 2013). The other three viruses (SARS-CoV, MERS-CoV, and SARS-CoV-2) cause severe respiratory disorders in humans and even death in severe cases (van der Hoek, 2007; de Groot et al., 2013). Bats typically feed on flying insects, prefer to live in caves, abandoned mines, tunnels, hollow trees, etc., and are widespread on the planet. There are over 1,300 bat species found worldwide that harbor several pathogenic viruses (including SARS-CoV and, MERS-CoV, SARS-CoV-2) that cause human diseases. Their gregarious living habits provide possible survival conditions and cell types for spreading zoonotic viral infections among birds, animals, and humans (Plowright et al., 2015). The ability of bats to migrate over long distances facilitates the exchange of viruses or their genetic material between bats and other animals, indicating that different living environments lead to the evolution of diverse CoVs. Over the last 20 years, several CoVs (SARS-CoV, MERS-CoV, SARS-CoV-2, etc.) have jumped between bats, animals, or humans, resulting in diseases with high fatality (Wong et al., 2021). Rodentia is one of the most diverse mammals, with over 2000 species carrying a wide range of microorganisms that are the primary source of infectious diseases. Many rodents live close to humans and pose significant zoonotic risks. Only one rodent species has been identified as carrying CoV, indicating that rodents are the reservoir for RNA viruses. Murine CoV (mCoV) is a well-studied CoV archetype (Cheever et al., 1949). Following the isolation of the first MHV from mice in 1949, a mutant sialodacryoadenitis-CoV (SDAV) was discovered in 1970 (members of the beta CoV) (Wang et al., 2015). Notably, rodent-associated CoVs account for most of the known HCoV-HKU1 and HCoV-OC43 genetic diversity, which causes enteric and respiratory diseases in humans and domestic animals (Grabherr et al., 2021). As a result, further research into the rodent’s potential role in the evolution of CoVs is required to unravel the mysteries of future viral outbreaks (Han et al., 2015). Notably, murine thrive in urban areas and are well-adapted to coexistence with wildlife and humans (Langer and Giessen, 2007; Bartak et al., 2021), and they may spread unidentified CoVs in the community. Such viruses include swine enteric CoVs (SeCoVs), Transmissible gastroenteritis virus (TGEV), porcine epidemic diarrhea virus (PEDV), etc. (Pan et al., 2017; Cheng et al., 2020; Li et al., 2022b). The porcine respiratory CoV (PRCV) and TGEV are closely related to the feline and canine CoVs (Liu and Wang, 2021). Porcine hemagglutinating encephalomyelitis virus (PHEV) infections are common in pigs, causing encephalomyelitis and severe vomiting in piglets (Liu and Wang, 2021). Porcine enteric alpha CoV (PEAV) was first discovered in southeast China in 2017 and shares 90% aa homology with bat CoV-HKU2 (Gong et al., 2017). Later, a PEAV was identified and isolated in China (Yong-Le et al.). PEDV and PDCoV are the most pathogenic porcine viruses, threatening the swine industry and causing watery diarrhea in piglets (Surapong et al., 2019). The coinfection of PDCoV and PEDV may result in higher piglet mortality. Moreover, CoVs cause respiratory, intestinal, and systemic diseases in many other hosts. Most clinical manifestations are mild, but a few can have serious public health consequences. In cattle and wild ruminants, Genera beta CoV causes respiratory and intestinal disorders (Vlasova and Saif, 2021). Avian infectious bronchitis is a contagious upper respiratory tract disease in chickens caused by gamma CoV (infectious bronchitis virus—IBV) that significantly impacts the poultry industry globally (Legnardi et al., 2020). Feline CoV (FeCoV) is well-known for causing minor intestinal infections in domestic cats (Oguzoglu et al., 2021). Canine respiratory CoV (CRCoV) causes respiratory diseases in dogs (Priestnall, 2020), whereas ferret entero-CoV (FrECoV), alpaca CoV, and equine CoV (ECoV) are pathogenic to humans and their companion animals (Csiszar et al., 2020; Gilmutdinov et al., 2020; Islam et al., 2021).

## CoVs cross-species transmission

The interaction between the virus and the host is the primary driving force behind virus evolution. A virus with a faster rate of evolution may be better suited for host and cross-species transmission. CoVs are sufficient to provide more base space for gene mutations. Mutations at the gene level can be used to adapt to the host body’s environment and jump to new hosts, promoting cross-species transmission and forming a wide host distribution (Olival et al., 2017). As stated earlier, bat species are the primary reservoirs for many pathogenic viruses, including CoVs. Hosts and viruses have either co-evolved or been paired through spillover, and ample studies show how reservoir hosts like bats have likely co-evolved with CoVs (Wessner, 2010). It has been established that initial CoV transmission occurs from bats, but the underlying transmission mechanism from these mammals to humans requires further investigation. Literature has shown how intermediate hosts played a crucial role in the transmission and evolution of CoVs and other related viruses (Graham and Baric, 2010; Cotten et al., 2013; Enserink, 2013).

The close identity of alpaca CoVs and HCoV-229E with African bat CoVs (Corman et al., 2015) implies that humans may have contact with these bats in their natural habitats rather than alpacas, which do not share habitats. Further investigation reveals that camelid CoVs shared genomic similarities with HCoV-229E, implying the evolution of a new viral genotype toward humans. A mutation (deletion/insertion) in the *S* gene sequence facilitates the transfer of bat-associated HCoV-229E to humans, establishing camelids as the primary zoonotic source for humans (Corman et al., 2018). Artiodactyls, carnivores, lagomorphs, perissodactyls, primates, and rodents have all been found to carry HCoV-OC43 (Mulabbi et al., 2021). The best representative of these CoVs are bovine CoVs, beta CoVs, and HCoV-OC43 reported in livestock, which might transmit to humans. The S gene mutation (deletion or insertion), like other CoVs, reflects HCoV-OC43 adaptation to the human environment. Like beta CoVs, HCoV-OC43 has an ancestral link to rodents (but not bats) due to their close similarity to MHV, indicating their origin in rodents (Corman et al., 2018). However, the transmission mechanism of HCoV-OC43 from rodents to bovines is unknown, whereas HCoV-NL63 is related to some bat-associated CoVs of the *Vespertillionidae* and *Hipposideridae* families (Pfefferle et al., 2009). So far, no zoonotic reservoir has been identified as the underlying transmission mechanism of HCoV-NL43 to humans. Moreover, there are no sequence similarities between rodent-associated HCoV-HKU1 and other animal CoVs (Woo et al., 2005; Wang et al., 2015). Like HCoV-NL63, the HCoV-HKU1 transmission mechanism from animals to humans is unknown. Further, rodent-associated HCoV-HKU1 shares no sequence similarities with other animal species (Woo et al., 2005; Wang et al., 2015). In light of the recent SARS-CoV, MERS-CoV, and SARS-CoV-2 outbreaks, new insights into CoV transmission patterns will require extensive research in future viral outbreaks (Guan et al., 2003).

Bats have been identified as a natural reservoir for an increasing number of emerging zoonotic viruses. A study identified the horseshoe bat (genus Rhinolophus) as the reservoir host for many viruses with a close genetic relationship to SARS-like COVs (SLCoV). Additional novel bat SLCoV detection has shed new light on the origin and transmission of various SARS among wild mammals (Wang et al., 2006). These animals were thought to harbor SARS-CoV in their markets, likely where the virus originated before causing human disease (Weiss and Navas-Martin, 2005; Zhou et al., 2020). Although SARS-CoV has been discovered to infect monkeys and domestic cats, there is no evidence of virus transmission from these animals to humans (Chan et al., 2013; Sabir et al., 2016), suggesting the virus originated in a wild, natural reservoir. Due to the sequence identity of MERS-CoV, with bat-CoV-HKU4 and KHU5, the first attempt focused on bats (Chan et al., 2013). According to the molecular and serological investigation, MERS was first reported in camels in Saudi Arabia, Oman, and Qatar (Chan et al., 2013; Mulabbi et al., 2021). In Saudi Arabia, dromedary camels harbor several CoV lineages, including the one responsible for human outbreaks. The infectious MERS-CoV strain isolated from these camels demonstrates the outbreak’s severity (Sabir et al., 2016). It was speculated that the close association of dromedary camels with humans might result in a novel HCoVs outbreak (as the current SARS-CoV-2 pandemic)—most likely due to human interactions with bats or other intermediate hosts (Wang et al., 2005; Baseler et al., 2016).

The SARS-CoV-2 outbreak in humans has been linked to China’s Wuhan Seafood Market, where several birds, rodents, and rabbits were sold (Mackenzie and Smith, 2020; Zhou et al., 2020). SARS-CoV was closely related to bats that originated SLCoV-ZC45 and SLCoV-ZXC21, which had previously been identified in China (Hu et al., 2018), indicating that bats are natural reservoirs for such viruses. SARS-CoV-2, like other HCoVs, is likely to transfer to an intermediate host in the same market before spreading to humans (Li et al., 2020b). This is because there is no evidence of human contact with the virus-carrying bats, which are known to hibernate in December (Li et al., 2020b; Shah et al., 2022). Next-generation sequencing revealed SARS-CoV’s resemblance with previously identified SLCoV-ZC45 and SLCoV-ZXC21 (Hu et al., 2018), revealing bats as their natural reservoir.

### CoVs human-to-human transmission

**Figure 2:**
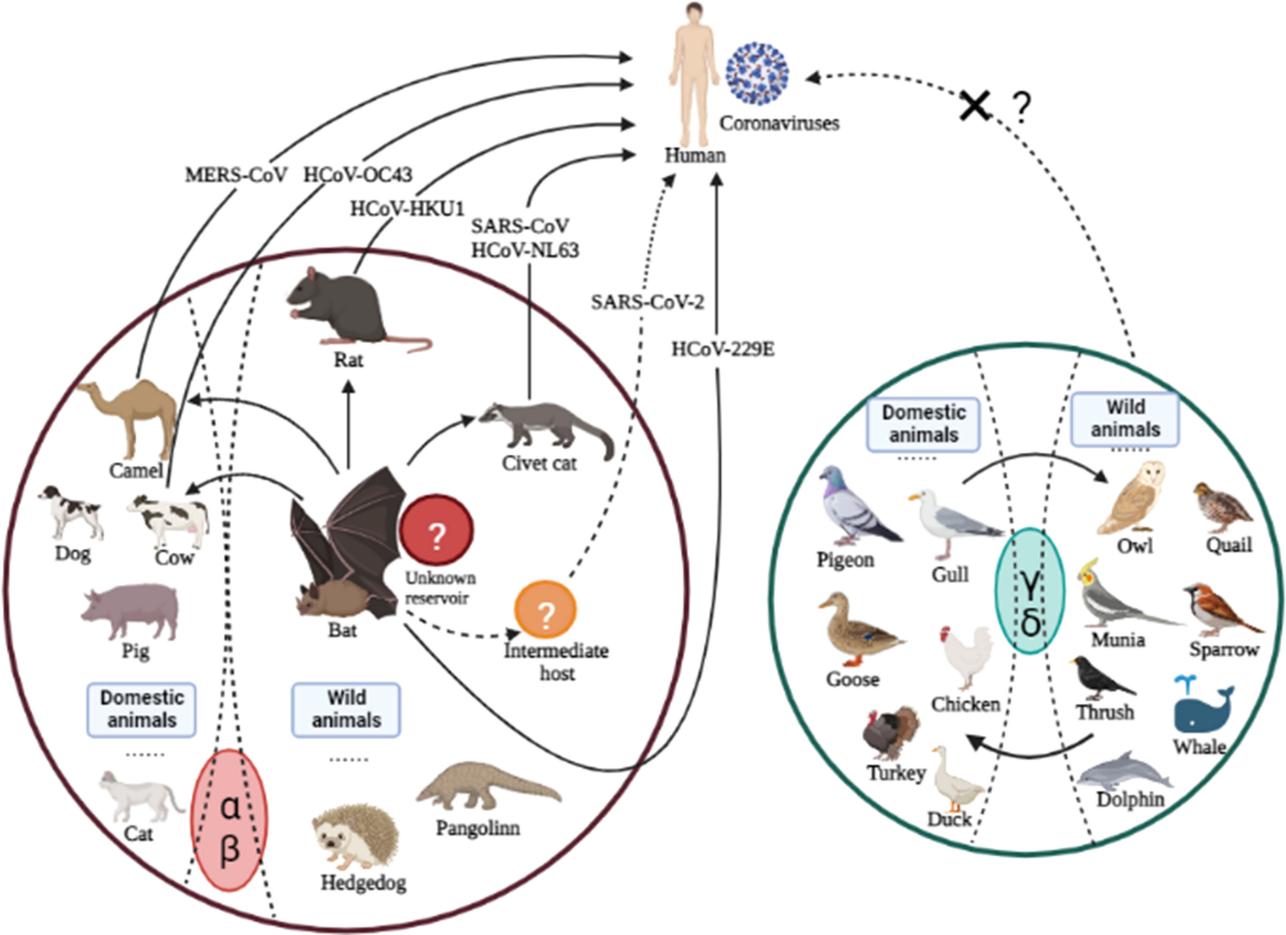
Cross-species transmission of CoVs is common among various animals. The arrows indicate the direction of virus transmission. The transmission sources of seven CoVs (all belonging to genera α and β) infecting humans are depicted are intragenus mutual transmission. There have been no reports of human infections with genera γ or δ, which calls for additional research in the future.

Once zoonotic CoVs cross the species barrier for human infections, their survival in humans depends on their ability to sustain transmission from person to person through aerosolized particles or physical contact with infected people or objects. The CoVs responsible for human outbreaks are HCoV-229E, HCoV-HKU1, HCoV-NL63, HCoV-OC43, SARS-CoV, MERS-CoV, and SARS-CoV-2, which spread via air droplets, direct contact with an infected person, or contaminated surfaces. The viruses primarily replicate in the respiratory tract and then spread to other body organs (Li et al., 2020bb; Mulabbi et al., 2021). Furthermore, virus transmission from infected individuals to healthy individuals depends on the virus’s stability and adaptation to a new host or environment. It has been reported that HCoV-NL63 can survive in an aqueous solution and the respiratory tract for at least seven days (Müller et al., 2008) at 20°C and 50% humidity (Chowell et al., 2015; Hunter et al., 2016). MERS-CoV can survive for 48 hours at 20°C (40% humidity), while SARS-CoV can survive for five days at 22°C (50% humidity). Both viruses lose viability when temperatures and humidity rise (van Doremalen et al., 2013). Because of this stability, viruses can be transmitted via aerosols; for example, SARS is more stable and has a longer transmission via aerosol than MERS. In addition, these viruses are stable on inanimate surfaces and infect humans after touching the contaminated surfaces (Lee and Wong, 2015), explaining human infections that were not close to patients during their respective outbreaks. SARS-CoV can be excreted in feces and remains infectious in sewerage for up to 48 hours, implying that viral shading in stool and clogged sewerage canals, as well as the possibility of fecal-oral transmission, may be a potential transmission route when people come into contact with contaminated sewerage (Wang et al., 2005; Young et al., 2020). The disposal of virus-containing waste in landfills can contaminate drinking water, necessitating the centralized disposal of environmentally sound units. SARS-CoV-2 can be transmitted from an infected person to a healthy person, as evidenced by infected families and medical staff who did not visit the market where the virus was primarily reported (Carlos et al., 2020; Wang et al., 2020a). The ability of the SARS-CoV-2 infection to remain alive on surfaces for days allows virus transmission and fomites (van Doremalen et al., 2020). In contrast to SARS and MERS, which cause intestinal infections at later stages; whereas, SARS-CoV-2-infected individuals may harbor the virus in their intestines at any stage of infection. The detection of SARS in the oral cavity, anus and body fluids demonstrates an alternative transmission route (Zhang et al., 2020), which suggests additional research to understand virus dynamics and transmission better. The cross-species transmission of some known CoVs among various animals is shown in **Figure 2**.

## Conclusion and future perspective

COV infection in humans causes mild to severe acute respiratory illnesses with high fatality rates. CoVs, like other emerging pathogens, pose a great public health challenge due to the lack of information before they jump from primary sources, i.e., wildlife to birds, humans, or other mammals.

Humans are encroaching on new habitats, and increasing their interactions with wildlife (and the diseases they carry) has serious public health implications. Diseases that spread from animals to humans are rising; for example, SARS-CoV spread from bats and civet cats, Spanish influenza affected birds and other mammals before transferring to humans, and SARS-CoV-2 spread from a wildlife market to humans. Thus, zoonotic spillover of viruses is always possible; thus, harmonious development can effectively prevent viral zoonosis by maintaining a barrier between human society and the virus’s natural host (Albery et al., 2021). The most significant challenge is developing preventive therapies and vaccines in time for new viral outbreaks. Since bats naturally harbor a variety of CoVs, ongoing efforts should be made to identify and characterize bat-associated viruses and the risk of transmission to domestic animals and humans. Identifying the viruses’ underlying cross-host species transmission mechanisms that link bats to humans is critical. Apart from the indirect transmission, there is always the possibility of virus transmission from bats to humans, which needs to be thoroughly investigated. In addition to identifying intermediate hosts, viral surveillance studies should be expanded to wildlife, birds, animals, and mammals before they spread to humans. Mutations (sequence deletion/insertion) and recombination in ssRNA viruses contribute to the emergence of novel pathogenic viruses capable of infecting humans. Consequently, ongoing monitoring of virus mutations is required to ensure that their emergence does not catch humans off guard, and preventive measures to prevent zoonotic viral transmission are recommended. Stringent regulations to monitor wildlife trading, barriers around wild mammals and animals, and a general shift in community cultural practices should all be monitored for this purpose. These can be supplemented with other commonly used methods, such as infected person isolation and restricting public mobility in CoV-endemic areas. The One Health concept is recommended for approaching and eradicating future public health risks from emerging novel viral outbreaks.

## Author contributions

QL and TS performed the initial draft preparation and revision. XX, TS, LQ, BW, RW, ZB, and YH made suggestions for the writing of the manuscript and revision. All authors actively contributed to the article and approved the submitted version.
